# Idiopathic chronic eosinophilic pneumonia

**DOI:** 10.1186/1750-1172-1-11

**Published:** 2006-04-06

**Authors:** Eric Marchand, Jean-François Cordier

**Affiliations:** 1 Service de Pneumologie, Cliniques Universitaires de Mont-Godinne, Université Catholique de Louvain, B-5530 Yvoir, Belgium; 2 Service de pneumologie-Centre des Maladies Orphelines Pulmonaires, Hôpital cardiovasculaire et pneumologique Louis Pradel, 28 Avenue du Doyen Lépine, 69677 Bron Cedex, France

## Abstract

Idiopathic chronic eosinophilic pneumonia (ICEP) is characterized by subacute or chronic respiratory and general symptoms, alveolar and/or blood eosinophilia, and peripheral pulmonary infiltrates on chest imaging. Eosinophilia is present in most cases, usually in excess of 1000/mm^3^. In absence of significant blood eosinophilia, a diagnosis of ICEP is supported by the demonstration of bronchoalveolar lavage eosinophilia. ICEP is typically associated with eosinophil counts higher than lymphocyte counts in the bronchoalveolar lavage. ICEP is a rare disorder of unknown cause. Its exact prevalence remains unknown. ICEP may affect every age group but is rare in childhood. It is twice as frequent in women as in men. One third to one half of the ICEP patients have a history of asthma. The mainstay of treatment of ICEP is systemic corticosteroids. Response to oral corticosteroid therapy is dramatic and has led to the consideration of corticosteroid challenge as a diagnostic test for ICEP. Nevertheless, relapses or development of severe asthma are frequent when tapering or withdrawing treatment. Long-term oral corticosteroid therapy is necessary in up to half of the patients.

## Disease name and synonyms

Idiopathic chronic eosinophilic pneumonia (ICEP);

Chronic eosinophilic pneumonia (CEP);

Carrington's disease.

## Diagnosis criteria/definition

Idiopathic chronic eosinophilic pneumonia (ICEP) is a rare disorder of unknown cause characterized by subacute or chronic respiratory and general symptoms, alveolar and/or blood eosinophilia, and peripheral pulmonary infiltrates on chest imaging. There are no strict diagnostic criteria for ICEP. Diagnosis is usually based on the association of:

1) respiratory symptoms of usually more than 2 weeks duration;

2) alveolar and/or blood eosinophilia (alveolar eosinophilia ≥ 40% at bronchoalveolar lavage (BAL) differential cell count; blood eosinophilia ≥ 1000/mm^3^);

3) pulmonary infiltrates with usually a peripheral predominance on chest imaging;

4) exclusion of any known cause of eosinophilic lung disease.

## Epidemiology

ICEP is a rare disorder. Its exact prevalence remains unknown. ICEP has been reported to contribute to 0–2.5% of cases included in different registries of interstitial lung diseases [1].

ICEP may affect every age group but is extremely rare in childhood [2-5]. It is twice as frequent in women as in men [2,3,6,7].

One third to one half of the ICEP patients have a history of asthma [7-12]; less than 10% are active smokers [2,3,7]. It has recently been reported that ICEP may be primed by radiation therapy for breast cancer [13].

## Clinical description

Symptoms are non-specific and usually include respiratory and general symptoms, which are either subacute or chronic. Accordingly, the symptoms are most often present for about a month before diagnosis is made.

### Respiratory signs

Dyspnea and cough are always present. The severity of dyspnea is highly variable [3]. Respiratory failure necessitating mechanical ventilation, however, is very rare [14,15], in contrast with idiopathic acute eosinophilic pneumonia. Wheezing occurs in about half of the cases. Chest auscultation findings are non-specific, with possible inspiratory crackles or expiratory wheezes [3].

### General manifestations

Asthenia, weight loss (sometimes marked), and nocturnal sweat or fever are frequent [2,3,7].

### Extrathoracic manifestations

Extrathoracic features are absent in ICEP. When the symptoms and signs of extra-pulmonary involvement are present, a diagnosis of Churg-Strauss syndrome (CSS) or idiopathic hypereosinophilic syndrome (IHS) should be considered. A few patients with an initial diagnosis of ICEP, however, may develop minor extrathoracic manifestations without further fulfilling the diagnostic criteria for either CSS or IHS. It is noteworthy that a continuum between ICEP and CSS has been suggested [16-18].

## Paraclinical findings/diagnostic studies

### Chest imaging

ICEP is characterized by peripheral parenchymal infiltrates on chest imaging. These infiltrates can be either unilateral or most often bilateral. The most distinctive chest X-ray images mimic the photographic negative of acute pulmonary edema [10]. This typical pattern, however, is absent in the majority of cases of ICEP [2] and is not specific for the disorder, since it has also been described in cryptogenic organizing pneumonia, sarcoidosis, or drug-induced pneumonia. The parenchymal infiltrates are alveolar in nature, ranging from ground-glass opacities to consolidation with air bronchogram. They are sometimes migratory [3,7]. Pleural effusion is uncommon.

Computing tomography (CT) may show discrete ground-glass opacities that are not discernible on X-rays. Bilateral parenchymal abnormalities are commonly seen with CT [3]. Mediastinal lymph node enlargement has been described [3,19].

### Laboratory findings

Although anecdotal cases without blood eosinophilia have been reported [2,9,20], it is present in most cases, usually in excess of 1000/mm^3 ^[3]. In the absence of significant blood eosinophilia, a diagnosis of ICEP is supported by the demonstration of bronchoalveolar lavage eosinophilia (≥ 40%). Erythrocyte sedimentation rate and C-reactive protein level are usually increased. Total immunoglobulin E (IgE) levels are elevated in about half of the cases [3], reflecting the high proportion of patients with an atopic background.

### Bronchoalveolar lavage

Bronchoalveolar lavage in ICEP always reveals abnormally high levels of eosinophils, representing 12% to 95% (mean: 58%) of the total cell count [3]. In contrast to cryptogenic organizing pneumonia with which it shares many common features, ICEP is typically associated with eosinophil counts higher than lymphocyte counts in BAL.

### Pulmonary function tests

ICEP can be associated with either a restrictive or an obstructive pattern on pulmonary function tests. The latter pattern is more often present in patients with a history of asthma [2,16]. However, spirometry remains within normal limits in up to one third of the cases [3]. Diffusion tests often show a reduced carbon monoxide (CO) transfer factor (DLCO); the CO transfer coefficient (KCO) is reduced in up to one quarter of the patients with ICEP [3]. Arterial blood gases usually demonstrate a mild to moderate hypoxemia [2,9,16].

### Pathological findings

Pathological confirmation of ICEP is usually not necessary to establish the diagnosis. When performed, lung biopsy shows an interstitial and alveolar inflammation with a clear-cut predominance of eosinophils [2,9]. Infiltration of pulmonary vessels with eosinophils may be observed but necrotising or granulomatous vasculitis are not present in ICEP [6]. Foci of organising pneumonia are frequently observed [2].

## Differential diagnosis

### Eosinophilic lung diseases of known cause

Several drugs may cause eosinophilic pneumonias. The most frequently involved drugs are nonsteroidal anti-inflammatory agents, including salicylates used in inflammatory bowel diseases (*e.g*. sulfasalazine) and antibiotics, including minocycline and cotrimoxazole. A detailed history with respect to drugs consumption is needed in order to exclude any potential pharmacological cause of eosinophilic pneumonia. Updated information on causative drugs is available [21,22].

Allergic broncho-pulmonary aspergillosis (ABPA) and other allergic broncho-pulmonary mycoses are characterized by type I and III immune reactions against *Aspergillus fumigatus *or other fungi. Characteristic features of ABPA are asthma, migratory pulmonary infiltrates associated with blood and alveolar eosinophilia, central bronchiectasis, high total IgE levels, positive immediate and late skin tests in response to the causative agent, as well as positive specific IgE and precipitins [23].

Some parasitic infections that are generally transient are associated with eosinophilia and pulmonary infiltrates. Causative agents are *Ascaris lumbricoides*, *Toxocara canis*,*Taenia saginata*, *Trichinella spiralis *and *Fasciola hepatica*. For patients living in tropical areas or with a history of travel in tropical areas, other parasites must be included in the search: *Strongyloides stercoralis*, *Wuchereria bancrofti*, *Brugia malayi*, *Schistosoma mansoni *and *Schistosoma haematobium*. Diagnosis relies on serologic tests and/or stool examination [24].

Other conditions such as malignancy (*e.g*. lung cancer) and other infections (*e.g*. tuberculosis) are exceptionally associated with eosinophilic lung disease.

### Idiopathic eosinophilic lung diseases

Idiopathic acute eosinophilic pneumonia (IAEP) differs from ICEP by the more rapid onset and the severity of its clinical presentation. The time period between the onset of symptoms and the diagnosis is usually inferior to 14 days [25,26]. In contrast to ICEP, an atopic background is usually absent and there is no sex preponderance. Mean age at presentation is about 30. Patients present with cough and dyspnea, which is often severe and associated with tachypnea. Chest pain of pleuritic character and fever are frequently observed. Hypoxemic respiratory failure is frequent at presentation, often requiring mechanical ventilation [25,26]. On imaging, IAEP is characterized by diffuse bilateral air-space or interstitial opacities without peripheral predominance. Pleural effusion is frequent and sometimes bilateral [25]. The white blood cell count rarely points towards the diagnosis at presentation, with the eosinophil count being lower than 500/mm^3 ^in 2/3 of the patients [25,26]. The BAL, however, is the key to the diagnosis [25-27], showing an average percentage of eosinophils of 40% in one series [25]. IAEP dramatically improves with or without corticosteroid therapy but, in contrast to ICEP, relapses are not described after tapering or withdrawing treatment in IAEP.

Churg-Strauss syndrome (CSS) is a systemic eosinophilic vasculitis which usually develops in patients with a long history of atopic disease including asthma and, most often, allergic rhinitis. Dramatic blood eosinophilia and infiltration of a variety of tissues with eosinophils which ensue, are eventually followed by a systemic vasculitis. The latter affects many organs including the heart (heart failure, acute coronary syndrome, pericarditis), the gastro-intestinal tract (abdominal pain, gastrointestinal bleeding or perforation), the skin (purpura, urticaria, nodules), and the nervous system (mononeuritis multiplex, central nervous system involvement). Pulmonary infiltrates, similar to those described in ICEP, are observed in only half of the cases at the time of diagnosis [28]. Antineutrophil cytoplasmic autoantibodies (ANCA) with a perinuclear pattern (p-ANCA) and an anti-myeloperoxydase specificity are found in about 50% of patients with CSS [29]. As already mentioned, it is likely that a continuum exists between ICEP and CSS, at least in some patients.

Idiopathic hypereosinophilic syndrome (IHS), a rare and heterogeneous disorder, has been defined by blood eosinophilia greater than 1500/mm^3 ^for more than 6 months, and signs or symptoms of organ damage related to eosinophilic infiltration [30]. Cardiac involvement including mural thrombosis, endocardial fibrosis, which lead to restrictive cardiomyopathy, is the most important complication of IHS and the major cause of morbidity and mortality [31]. Pulmonary involvement is observed in 40% of cases and is characterized by interstitial infiltrates on chest imaging; pleural effusion is seen in 50% of affected patients [32].

Cryptogenic organizing pneumonia shares many features with ICEP with respect to clinical presentation and imaging [33]. However, it is not associated with blood eosinophilia and lymphocytes outnumber eosinophils in BAL.

## Treatment

Treatment of ICEP is based on oral corticosteroids. After initiation of treatment, symptoms as well as blood eosinophilia regress within a few hours and chest imaging results normalize within a few days [3]. This dramatic response to therapy has led to the consideration of corticosteroid challenge as a diagnostic test for ICEP [9]. There is no consensus, however, on the doses or the duration of the corticosteroid therapy. Most authors recommend initial doses of prednisone between 0.5 and 1 mg/kg/day and a gradual tapering of the dose for a total treatment duration of 6 to 12 months.

## Long-term outcome

Although response to corticosteroid treatment is dramatic and treatment always leads to complete resolution, relapses of ICEP are observed in up to 50% of patients [3]. These relapses occur while tapering the dose of corticosteroids or after weaning. Relapses remain as responsive to corticosteroids as the inaugural episode. Inhaled corticosteroids have been proposed in order to prevent relapses [7]. This is supported by a lower rate of ICEP relapse in asthmatics treated with inhaled corticosteroids [8].

The development of asthma in the follow-up of ICEP patients is a common finding [8]. Up to one third of the patients have asthma requiring long-term corticosteroid therapy after a diagnosis of ICEP has been made, and some patients develop a fixed obstructive pattern on pulmonary function tests [8]. Overall, more than half of patients affected by ICEP may require long-term oral corticosteroid therapy due to either multiple relapses or severe asthma [8]. It is thus advisable to recommend measures to prevent corticosteroid-induced osteoporosis from the start of the treatment.

## Unresolved questions

• Role of inhaled corticosteroids in the prevention of relapses of ICEP.

• Possible overlap or continuum between ICEP and CSS.

• Doses and duration of corticosteroid treatment.
